# Implications and Lessons From the Introduction of Genome-Edited Food Products in Japan

**DOI:** 10.3389/fgeed.2022.899154

**Published:** 2022-06-21

**Authors:** Makiko Matsuo, Masashi Tachikawa

**Affiliations:** ^1^ Graduate School of Public Policy, University of Tokyo, Tokyo, Japan; ^2^ Graduate School of Environmental Studies, Nagoya University, Nagoya, Japan

**Keywords:** genome-editing, food policy, regulation, governance, genetically modified foods, biotechnology, Japan

## Abstract

Japan clarified its regulatory approaches for products derived from genome editing technologies in 2019. Consequently, Japan has become a pioneer in the social implementation of such technologies, as to date, the notification process for three products, GABA-enriched tomato, fleshier red sea bream, and high-growth tiger puffer, has been completed. However, this has led to questions about how this was achieved, given the poor consumer acceptance and low public support for genetically modified (GM) foods in the past. This paper describes Japan’s regulatory approaches and their implementation guidelines for products created using genome editing technologies. It explains the governance of genome editing technologies and how the derived products have been introduced into society. The three factors that made this possible include: 1) improved R&D environments as a result of government-led innovation policy and regulations which have sought a balance between science and social demand 2) changes in the players (i.e. university startups), that engage in R&D and the strategies used for social introduction, and 3) social value changes—the recent rise in momentum for sustainable development goals (SDGs) and environmental, social, and governance (ESG) investing. The lessons and challenges in terms of R&D policy development and regulation from these analyses are presented. As the market size and social impact of genome-edited food products is limited, it is too early to fully assess this topic for Japan and thus, the analysis in this study is preliminary and must be revisited in the coming years.

## 1 Introduction

### 1.1 Background

Genome editing refers to the use of various technologies to modify a target base sequence in the genome of a living organism. Specifically, these technologies allow scientists to induce insertions into and deletions of genomic DNA and other minor changes at specific target locations and the induction of base replacements or deletions, as cuts are naturally repaired. This method can be used to make genetic changes that are equivalent to those achieved with conventional mutation strategies. Furthermore, gene inserts into target base sequences can be used to create modifications comparable to those achieved with genetic modification technologies. However, unlike conventional genetic modification technologies, which are imprecise as they do not allow insertions at specific sites, genome editing is precise and versatile; furthermore, less time is required for research and development (R&D). Consequently, the use of genome editing has rapidly expanded to include a more extensive range of targets (animals, plants, microorganisms, etc.) and applications (medical, industrial, agricultural/food, etc.).

There are ongoing discussions globally regarding the regulatory approaches for genome editing technologies (Lusser and Rodríguez-Cerezo, 2012; UK House of Commons Science and Technology Committee, 2015; Sprink et al., 2016; Wolt et al., 2016; Duensing et al., 2018; Dederer and Hamburger, 2019; Eckerstorfer et al., 2019; Special issue by Transgenic Research, 2019; Entine et al., 2021; Menz et al., 2020; Turnbull et al., 2021). Several countries have already developed legal frameworks based on conventional genetic modification technologies. However, there is a debate about existing laws and regulations and whether they should apply to products created using newer technologies, including concerns as to whether they accurately assess safety and appropriate management. The primary issue is whether genome editing technology products that do not contain any genes from other species and are indistinguishable from those derived from natural mutations or conventional breeding should be subjected to the same regulatory oversights applied to genetically modified (GM) products that contain genes from other species.

The authorities in Japan discussed their regulatory approaches for products derived from genome editing technologies in 2018 and clarified their guidelines in 2019 (Tsuda et al., 2019; Matsuo, 2021). All countries have different regulatory approaches for genome editing in relation to plants, animals, and microorganisms. Regulatory frameworks may differ depending on the intended use such as for food, or environmental release. Furthermore, relatively few countries have clarified specific regulatory guidelines for all products. Japan was one of the fastest countries to address regulatory clarification after such country as Argentina. Clarification of these regulatory approaches paved the way for the societal introduction of products derived from genome editing technologies. Before this, the only known genome-edited product available on the market was high-oleic soybean oil from the US company, Calyxt, Inc.^1^ At the time of writing (March 2022), Japan has completed the notification process for three food products, GABA-enriched tomato, fleshier red sea bream, and high-growth tiger puffer. Furthermore, two of the three products involved fish, which were expected to pose a more substantial challenge regarding public acceptance compared to plants.

### 1.2 Objectives of This Study

Existing literature has indicated that regulatory oversight systems often fall behind when an emerging technology is introduced to society (Marchant et al., 2011). It can be challenging to manage risks when legislative systems are structured in a path-dependent manner but a technology is in its earliest stages and thus its future direction and applications uncertain. In addition, survey results on the social acceptance of GM and genome-edited products, particularly for animals, which will be discussed in the following sections, suggest that ensuring public endorsement of animal products would be more complex than with plants. Therefore, the introduction of these products to society is expected to take time (Frewer, 2017; Tachikawa et al., 2017; Tachikawa et al., 2020; Busch et al., 2022).

Japan, however, has seen a relatively smooth societal introduction of these technologies. This was an unexpected phenomenon, given the country’s failure to promote previous genetic modification techniques in the food and agricultural sector, as they were characterized as having poor consumer acceptance^2^ and consequently there was almost no commercial cultivation of GM crops. This paper examines possible factors that enable genome-editing technologies to enter society, draws implications and lessons on governance for their societal introduction, and provides insights for countries considering the use of such products.

This study analyzed literature review results (including primary and secondary source materials) and interviews with domestic and international stakeholders (including public authorities, scientists, and industry sources).

## 2 Clarification Process for Regulatory Approaches and Commercialization

### 2.1 Growing Expectations for Biotechnology and Clarification of the Regulatory Guidelines in Japan.

#### 2.1.1 Policies Promoting Biotechnology Innovation

After 2018, the Japanese government issued several policy documents concerning biotechnology, which spurred the development and application of genome editing technologies and efforts to clarify the required regulatory approaches. The Integrated Innovation Strategy,^3^ which the Cabinet approved in 2018, required clarification of the regulations for products derived from genome editing technologies. In the following year’s edition of the Integrated Innovation Strategy, biotechnology was selected as one of the three fields of focus, along with artificial intelligence (AI) and quantum technologies. Bio Strategy 2019 was announced in the same year, and this was the first update to the strategy in 11 years.^4^ These government strategies called for clarification of the regulatory approaches for products derived from genome editing technologies and their impacts on biodiversity and food safety. Government ministries were prompted to start discussions on relevant regulatory issues. In February 2019, the Ministry of the Environment (MOE) issued a notification^5^ concerning its approaches to biodiversity in relation to products derived from genome editing technologies, while the Ministry of Health, Labour, and Welfare (MHLW) issued food safety handling procedures^6^ in September of the same year. This effectively completed the legal framework for the application of genome editing technologies in Japan, establishing systems for providing information and prior consultation/notification with the relevant authorities.

#### 2.1.2 Regulation Clarification: Impacts on Biodiversity

**FIGURE 1:**
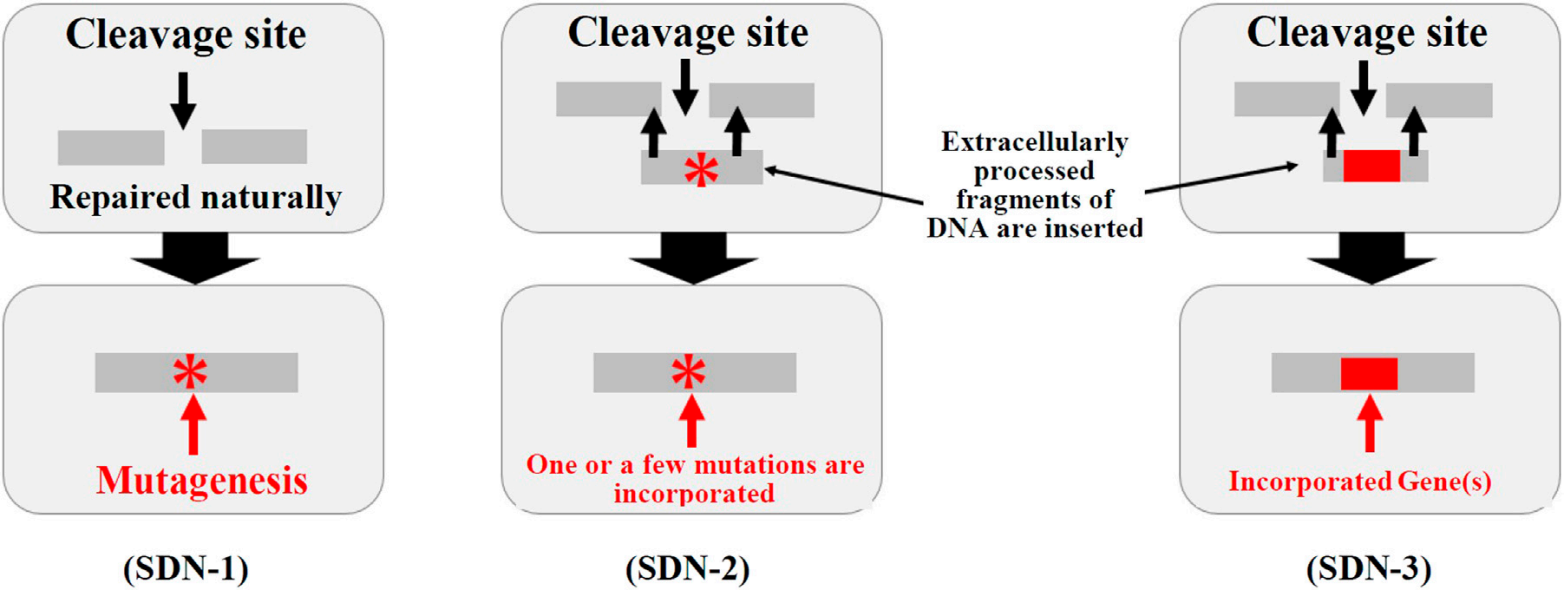
Ministry of the environment classification of SDN-1, -2, and -3. (Source) Created by the authors based on the Technical Committee on Genetically Modified Organisms. “Regulatory status and handling policy of organisms obtained using genome editing technologies under the Cartagena Act (draft),” 30 August 2018, pp. 2, available on the Ministry of the Environment website https://www.env.go.jp/council/12nature/y120-37b/13mat4-3.pdf (accessed 17 March 2022).

In Japan, biodiversity-related considerations for modified living organisms are managed under the so-called Cartagena Act,^7^ and the law requires an assessment of their impacts on biodiversity. The MOE discussed whether living organisms created using genome editing technologies should be subject to the Cartagena Act. Genome editing allows scientists to develop a variety of genome editing-derived organisms using site-directed nucleases (SDNs) (See Figure 1). SDN-1 is defined as the technique of introducing a break at a target site in the genome to cause a loss of gene function through “errors” that may occur during the natural repair process. SDN-2 involves the addition of a template to the target site to cause a few base pairs to be modified, whereas SDN-3 introduces a gene or genes at the target site. Using the definitions in the Cartagena Act, the MOE has determined that Living Modified Organisms (LMOs) are not subject to regulation if 1) they do not contain DNA or RNA that was processed outside the cell or if 2) the introduced DNA or RNA is no longer present in the final organisms—such organisms are known as null segregants. In contrast, LMOs harboring introduced extracellularly processed nucleic acids are regulated. In other words, SDN-1 organisms are not subject to regulation under the Cartagena Act, whereas those created using SDN-2 and SDN-3, which involve templates and the insertion of a gene or genes, respectively, are subject to regulation. Even when a product is deemed not subject to law, its developer is asked to provide relevant information to the respective government agencies in advance. A summary of such information is made available to the public on the MOE’s Japan Biosafety Clearing-House (J-BCH) website.^8^ In response to the clarification made by the MOE, the government agencies developed handling policies for each usage. In October 2019, the Director-General of Food Safety and Consumer Affairs Bureau (FSCAB/MAFF) issued a procedure for providing information on the impacts of agricultural products on biodiversity ^9^.

#### 2.1.3 Regulation Clarification: Food Safety and Labeling

To assess the food safety aspects of GM products, Japan requires safety assessments under the Food Sanitation Act (Act No. 233 of 1947) and Food Safety Basic Act (Act No. 48 of 2003).^10^ The MHLW—the government ministry responsible for the food safety aspects of GM products (food and food additives)—stipulated that the application of GM food regulations should be determined based on whether the specificity and range of the mutations in the foods derived from genome editing technologies are comparable in terms of safety to those of natural mutations or mutations that occur in conventional breeding (such as induced mutations using chemicals), which are not subject to GM food regulations. This means that GM food regulations would apply to SDN-3 products that contain transgenes but not to SDN-1 products that do not involve transgenes, whereas decisions on SDN-2 products would be made on a case-by-case basis. Developers of food products derived from genome editing technologies are asked to have prior consultation with the MHLW and are expected to complete the notification process with the ministry if the product is not subject to regulation before distribution^11^. Once the notification process has been completed, a summary of the information will appear on the MHLW website^12^ ^,^ ^13^.

Under the JAS Act and Food Sanitation Act, GM foods must be labeled to allow consumers to make their own choices.^14^ The Consumer Affairs Agency (CAA), which is responsible for food labeling, clarified that those foods derived from genome editing technologies which are not subject to regulation as a result of the prior consultation are exempt from labeling.^15^ At the same time, specific considerations were provided in response to demands for the consumer’s right-to-choose. It was recommended that businesses should make an effort to share information on their food products if they are known to be derived from genome editing technologies by means including labeling (based on reasonable evidence, including transaction records from the food supply chain), even when they are not subject to regulation.^16^ However, the CAA does not specify how the information should be provided to the consumers.

### 2.2 Notifications for Products Derived from Genome Editing Technologies, their Market Launch, and Public Response

#### 2.2.1 Notified Products Derived From Genome Editing Technologies for Food Use

Following the clarification of the regulatory approaches for products derived from genome editing technologies, the notification process for food use has been completed for three food products (at the time of writing—March 2022). The three products are as follows:

##### 2.2.1.1 GABA-Enriched Tomato

Notification for this product was filed with the MHLW, and information was submitted to the Ministry of Agriculture, Forestry, and Fisheries (MAFF) on 11 December 2020, by Sanatech Seed Co., Ltd. The product contains GABA, a compound found in tomato fruits that aids relaxation and lowers blood pressure, at a level 4–5 times higher than that of a regular tomato.^17^ In May 2021, the company provided GABA-enriched tomato seedlings to home gardeners at no cost. Then in September of that year, they started selling the tomato fruits online, and in October the seedlings for home gardeners.^18^

##### 2.2.1.2 Fleshier Red Sea Bream

Notification for this product was filed with the MHLW, and information submitted to the MAFF on 17 September 2021, by Regional Fish Institute, Ltd. In this product, the myostatin gene, which suppresses muscle growth, was made dysfunctional, leading to an increased proportion of muscle tissue. The company started fundraising for the product under the name “22nd Century Sea Bream” using a crowdfunding platform. They raised over 3.2 million yen, which was more than their stated target of 1 million yen. The crowd funder’s rewards include fish products that began shipping in October 2021.^19^

##### 2.2.1.3 High-Growth Tiger Puffer

Notification for this product was filed with the MHLW, and information was submitted to the MAFF on 29 October 2021, by Regional Fish Institute, Ltd. By disabling the hormone leptin,^20^ which controls appetite, scientists succeeded in making the fish grow 1.9 times larger and improved the efficiency of feed use (the weight gained by the animal divided by the weight of feed intake) by 42%.^21^ The company fundraised for the product under the name “22nd Century Tiger Puffer” using a crowdfunding platform^22^. This campaign raised almost 3.9 million yen, which was higher than the original target of 1 million yen.

In December 2021, Regional Fish Institute Ltd. began selling both the “22nd Century Sea Bream” and “22nd Century Tiger Puffer,” online through its website^23^.

#### 2.2.2 Gap Between the Anticipated and Actual Public Response

Given the low public acceptance of GM in Japan, it was anticipated that the societal introduction of genome editing technologies would face a degree of public controversy. A previous consumer perception survey (Tachikawa et al., 2017) found more support for tight regulations of genome-editing-derived foods which were designed to reduce the risk to as close to zero as possible rather than scientifically proven regulations and technically reasonable^24^. Since the regulatory clarifications in Japan exclude some genome editing-derived products from regulation (as described in Section 2.1. above), it was expected that consumers would demand regulations that are more stringent. In terms of application, previous studies and surveys on GM have suggested that products valued by producers are less likely to be accepted if consumers do not find them beneficial (Siegrist, 2008; Frewer, 2017). Similar results were observed with the promotion of genome editing. For example, a survey conducted by Saito (2021) found that “the public is more inclined to support the promotion of such technology when it has direct and clear benefits to consumers.” The application of technologies on animals, in particular, has been commonly found to meet greater resistance (Frewer, 2017; Busch et al., 2022). Therefore, it was considered that obstacles to public acceptance would be crucial for the two cases discussed that involve fish. For instance, Tachikawa et al. (2020) reported that, where genome-editing technologies are concerned, respondents were more opposed to using these technologies to increase the size of livestock by 50% (increased by a factor of 1.5) than for the development of vegetables or livestock with improved disease resistance. The previous GM food controversies from the late 1990s, which were driven by a number of major news companies and consumer group coalitions, questioning their environmental and food safety risk (Yamaguchi and Suda, 2010) exhorted the Japanese government to implement a mandatory legal authorization scheme for GM products. It was thus anticipated that the introduction of newer technologies would likely meet strong resistance. However, even though there were indeed some social actions, for instance, some groups of coop were against the use of genome-editing; petitions were made by some consumer groups;^25^ they did not develop into a mass mobilization,^26^ and media coverage was mostly positive. After filing the notifications, there were no considerable public reactions, nor did they receive any sustained attention.

Following the completion of the notification process, the Sanatech Seed Co., Ltd., which developed the GABA-enriched tomato, launched a campaign at the end of December 2020 to provide its seedlings to home gardeners at no cost. The 5,000 free samples were all claimed within a short period, and the company started shipping the seedlings in May 2021 (the home-grown tomatoes would be ready to harvest June)^27^.

## 3 Factors That Facilitated the Societal Introduction of Products Derived From Genome Editing Technologies

Questions resulted from the relatively smooth introduction of the three previously mentioned genome editing-derived products in Japan, as historically GM products have led to public controversy. Three potential factors and explanations are presented below.

### 3.1 Improved R&D Environments and Regulatory Approaches

Some of the fundamental factors may include the following: 1) improved government-led policy and research funds for the R&D of these technologies, and 2) the regulatory approaches that were made clear, with a certain degree of consideration given to both scientific discussion and public demand.

#### 3.1.1 Favorable Condition for R&D Led by the Japanese Government

Promoting a broad range of state-level projects improved the environment for R&D. Notably are projects such as the SIP (Cross-ministerial Strategic Innovation Promotion Program), JST OPERA (Program on Open Innovation Platform with Enterprises, Research Institute and Academia), and PRISM (Public/Private R&D Investment Strategic Expansion Program). Many of these government-led mission-oriented projects did not limit themselves to R&D activities but also aimed at the social implementation of their technologies, which increased momentum for their commercialization. In the SIP, for instance, both 1st and 2nd-period programs are designed within 5 years, and researchers are instructed to complete the social implementation of their research in this short research period. This has given rise to university startups. For instance, the aforementioned GABA-enriched tomato, created by the University of Tsukuba, originated from the first SIP project. In the 2nd SIP period, a website was created to disseminate information and increase the public’s understanding in support of genome-editing and its products.^28^

A major issue that arises regarding the improvement of the environment for developing and using technologies is the use of patents. CRISPR/Cas9 has been at the center of a dispute over the use of a patent, giving rise to concerns over uncertainty by potential users. However, uncertainty is limited in the agricultural field, as Corteva Agriscience and the Broad Institute have a unified contact point for patent licensing.^29^ This may also have been a crucial factor contributing to the rapid social implementation of the GABA-enriched tomato’s commercial application which used CRISPR/Cas9 technology.

#### 3.1.2 Regulatory Approaches That Aim to Strike a Balance Between Scientific Discussion and Public Demand

The commercial application of genome-editing technologies raises the issue as to how products should be controlled in the regulatory framework. The introduction of genome editing technologies in Japan was facilitated because of the following factors: 1) all aspects of the regulations for products derived from genome editing technologies, including the assessment of their impact on the environment and food safety issues, were made clear after the presentation of the cabinet-level policy in innovation and biotechnology, and 2) the regulatory approaches sought a degree of consideration for balancing scientific discussion and public demand.^30^

To elaborate on the latter, products derived from genome editing technologies that were excluded from the scope of regulation^31^ were harmonious with the thoughts of the scientific community to some extent, unlike the approaches taken in the European Union ^32^ and New Zealand (Fritsche et al., 2018), where all products derived from genome editing technologies are handled as GMOs. This may have been possible due to the limited scope of GM defined in our legislation. Japan defines GM products by the use of technology and excludes conventional breeding and mutagenesis from the outset. Japan has also introduced a system of providing information and notification, although it is not a legal obligation. It allows the regulatory authorities to review a product derived from genome editing technology before its use, determine whether it is subject to regulation, and collect a certain level of information on these products, even those that are deemed be exempt from regulation. This also helps, to some degree, to meet the concerns of people who demanded that the same levels of strict regulations as those for GM products be applied to products derived from genome editing technologies. In addition, the authorities will provide the public with a summary of the products confirmed to be exempt from regulation^33^ which secures a level of transparency. These systems represent approaches different from those of other countries, such as Australia and Argentina (Matsuo and Tachikawa, 2020). In Australia, the Office of the Gene Technology Regulator (OGTR) – responsible for the environmental assessment of GMOs, employs a similar scope for the environmental release of genome editing to that of Japan, however, it does not collect information on unregulated products. Like Japan, Argentina requires prior consultation but does not make the information on products derived from genome editing technologies public if they are exempt from regulation. Furthermore, while there is no legal requirement for the labeling of products derived from genome editing technologies that are not subject to regulation, Japan accommodates consumer sentiment and encourages businesses to share information on such products, wherever possible, to ascertain that it is derived with the use of a genome editing technology. No other country has taken such measures about labeling genome-edited products.

### 3.2 Changes to the Players Who Develop and Commercialize Technologies, Differences in Societal Introductions, and Changes to the Business Models and Strategies

The factors that helped to achieve the relatively successful introduction of genome-editing technologies may include a change in the players who develop or commercialize them and changes in the business models and strategies. Traditionally, the most widely used business model for GM products was Business to Business (B-to-B), where major GM multinational companies would develop technologies for key crops such as soybeans and corn, have them grown on a large scale by farmers and distribute them through wholesalers and retailers. The products derived from genome editing technologies introduced in Japan, in contrast, were developed by scientists at Japanese universities, marketed based on the Direct to Consumer (D-to-C) model, in which university startups delivered their products directly to consumers. In other words, these are niche products that were developed on a small scale. They are delivered directly to people who want them—the strategy in pursuit of product expansion is in an incremental manner.

In addition, these businesses have deliberately targeted sales strategies and pay close attention to consumer attitudes. Instead of putting their products through traditional distribution channels, they use the Internet and crowdfunding to sell to those interested in a manner that invites people to learn about their products and see the faces and ideas of their developers. The products of Sanatech Seed, Ltd. are sold only online and with a premium (higher price).^34^ Regional Fish also sell their products online. They can be bought by any consumer but the company states that it only sells to those restaurants who are in compliance with Regional fish’s transparency and traceability policy. The products that are sold online are fish for food (fresh fillet for Japanese hot pot, processed fried fish). As mentioned earlier, they also used a crowdfunding platform to inform people about the technologies and what the developers think and feel about the products, including how their products could contribute to the local fish industry. As rewards for supporters, they offered boxes of fish dishes featuring their product derived from genome editing technology.

To share information to enhance public understanding and acceptance, academia and businesses jointly engage in establishing initiatives^35^. In addition, rather than trying to gain support for non-labeling, there is willingness on the side of the producer to actively disclose the application of genome-editing technology to their products through voluntary labeling and traceability. As discussed above, Japan does not require the labeling of products derived from genome editing technologies if they are not classified as GM foods. Businesses are encouraged, however, to affirm information sharing on their products through labeling or other means if they are known to involve a product for which a notification has been filed. Sanatech Seed asserts that it will label tomato fruits and processed products (such as puree) made using genome editing technology.^36^ Similarly, Regional Fish has explained that it will ensure appropriate labeling and traceability and sell its products to partner businesses that can commit themselves to adhere to its labeling and sales policies.^37^ The other point regarding business is that the sources of Regional Fish finance include venture capitals and large investors, and regional banks.^38^ Management is optimized with the appointment of individuals with a background in government administration to utilize their knowledge and expertise relevant to new products. Regional Fish, for instance, has a bureaucrat sent by the MAFF using a recently established framework called “rental transfers” in which, personnel work for another company for a set period of time while remaining with the original organization.^39^ Its external auditor is a former administrator and has professional experience dealing with food safety.^40^

### 3.3 Other Possible Factors

Other factors contributing to the societal introduction of genome-editing technologies may include shifts in social values^41^. In the past, consumers were reluctant to accept GM crops. A common explanation for this was that GM crops were developed for the benefit of the producers. Traits such as tolerance to herbicides and resistance to pests were seen as beneficial for the producers and not for the consumers. However, people’s values may change, as suggested by the recent rise in momentum towards Sustainable Development Goals (SDGs) and Environmental, Social and Governance (ESG) investing. For instance, animals engineered to grow faster will benefit the producers. It may also be acceptable for consumers if it means efficient livestock fattening with a reduced environmental burden. On the food-tech front, the establishment of the Council for Public-Private Partnerships in Food Technology^42^ and its working team on smart breeding is one of the indications that society may be ready to embrace a sustainable food system based on new technologies.

## 4 Conclusion and Perspectives

Japan clarified its regulatory approaches for products derived from genome editing technologies in 2019. With the notification process completed for three such products, namely “GABA-enriched tomato,” “fleshier red sea bream,” and “high-growth tiger puffer,” Japan has become one of the pioneers in the social implementation of genome editing technologies. This paper outlines Japan’s clarification of regulatory approaches and the products for which notifications have been completed. In addition, it provided a preliminary explanation of possible factors that have enabled these technologies to be introduced into society in a relatively smooth manner.

The paper identified three factors. The first includes improving the R&D environment and clarifying the regulatory approaches. The environment for R&D contributed as 1) the government promoted R&D projects that emphasized the social implementation of the technologies and the sharing of relevant information with the public, resulting in the creation of several university startups.^43^ In agricultural field, in particular, the reduced uncertainty over the use of patents may also have contributed. 2) How the regulatory approaches were clarified was notable since scientific discussion and public demand was considered. Products that were not subject to regulation were aligned with the opinions of the scientific community. The regulatory agencies developed systems for collecting and providing information to monitor and accommodated public demand and consumer sentiment. They also tried to ensure transparency by sharing a summary of the information and notification on their websites. In addition, businesses are encouraged to share information on products that are exempt from labeling to respect consumers’ right to know. The second factor includes a player who develops the technologies and changes in business models and strategies. Unlike past approaches, where multinational companies distributed their products through general distribution channels on a large scale, current producers are startups from Japanese universities who engage with small niche targets. They deliver their products directly to consumers based on the D-to-C model while considering labeling and traceability. Furthermore, utilizing the expertise and experience of former/present government officials, may also contribute to the successful notification process and commercialization. The third possible factor involves a larger societal context: social value shifts. The recent increase in public awareness of the SDGs and expectations for food technology may have accelerated the acceptance of technologies that are presented in the context of reducing environmental loads and contributing to society.

Implications from these analyses on national policymaking for the application of genome editing technologies are that government authorities should endeavor to improve R&D strategies that incorporate social implementation within their scope. In doing so, it is also essential to have a mechanism that supports such application, as university researchers are usually not well equipped with business expertise. Another point is to clarify regulations by incorporating measures that provide a certain degree of consideration to public opinion (focusing on transparency and information disclosure in particular) while using the scientific discussion as a base. We need a mechanism by which to collect the information from products applying emerging technologies at the early stages of their introduction into society, to accumulate knowledge and respond to the need for transparency and informed choice. It may be unwise to reject these simply because it is not scientific or burdensome. Instead, this should be considered as a future investment for social acceptance. It is recommended that those who develop and commercialize these technologies should deliver their products to those who want them to meet public demand and ensure consumer choice, rather than selling on a large scale through general distribution channels at the initial stages. Another factor to consider is how the technologies are presented (i.e., positioned in the social context).

At the same time, it should be noted that it is too early to assess the Japanese case. The aforementioned genome-edited products for which the notification process has been completed have a limited impact. None of these products are found on the shelves of regular stores. These products begin and end within a small stratum of people who are interested in them. Although no technology will be accepted unanimously in society, another debatable point is whether one could introduce a technology only to those who embrace it. When a product derived from genome editing technology is commercialized in a crop intended for mass production, such as rice, it may face the real test in the societal introduction paradigm.

Finally, although the issues posed by different regulations in different jurisdictions were not the main subject of this study, this issue must be addressed in future research. There have already been many important contributions made to describe the different approaches taken in different countries and jurisdictions (Lusser & Rodríguez-Cerezo, 2012; UK House of Commons Science and Technology Committee, 2015; Sprink et al., 2016; Wolt et al., 2016; Duensing et al., 2018; Dederer & Hamburger, 2019; Eckerstorfer et al., 2019; Special issue by Transgenic Research 2019; Entine et al., 2021, Menz et al., 2020, Tuenbull et al., 2021). However, a detailed comparative analysis of the regulation of genome-edited products is urgently needed (Matsuo and Tachikawa, 2020), especially for topics such as on what grounds exemptions are made, what kind of information is gathered by the government and disclosed to the public, the impacts of the regulatory approach/style on innovation, relationship between R&D and the increase in the applied product, impact on industrial structure, as well as the policy formulation process (comparison with the previous GM policy development process). As mentioned briefly in this paper, how countries treat these products is different from jurisdiction to jurisdiction. A slight difference in regulation can have tremendous implications on international trade. GM regulations in each jurisdiction are already different, and the various ways of managing genome editing are widening those differences further. The prospect for international harmonization appears to be increasingly difficult. However, there might be room for international cooperation, particularly in information sharing and regulatory science. States may not support a common approach in regulating genome-edited products (i.e. risk management) but may and should be able to come to a common approach in assessing the safety of such products (i.e. safety assessment). The International Organizations that played essential roles in developing safety assessments of GMOs, such as OECD, CBD, and Codex Alimentarius Commission, should engage in horizon scanning activities, including information sharing of future biotechnology and work to elaborate on approaches to assess those products. In addition, developers and researchers are encouraged to actively participate in this process and create a shared understanding of the “responsible use” of these technologies’ and ELSI (Ethical, Legal and Social Implications/Issues).

Disclaimer: This analysis is an interpretation by the authors based on documents and information (sources include bulletins from government agencies, company websites, and the media) available at the time of writing. Many original sources referred to in this paper are in Japanese and were translated by the authors, as most of them did not have an official translation. Please refer to the official documents/organizations for interpretation and judgment. The authors take no responsibility for the translations and interpretation of the regulations.

## Footnotes

1 It is known that there are some genome-editing-derived products that are not subject to regulation in Argentina. However, since the specifics of such products have not been made public (Whelan and Lema, 2019), it is not possible to ascertain how many such products are exempted or commercially available.

2 For example, in a Consumer Affairs Agency survey on consumer attitude toward GM food, approximately 40% of the respondents said they were “concerned” about the safety of GM food. Consumer Affairs Agency (2017) 1st Study Group on Genetically Modified Labeling System in Japanese. Consumer Affairs Agency website (in Japanese) http://www.caa.go.jp/policies/policy/food_labeling/other/genetically_modified_food.html (accessed March 17, 2022).

3 “Integrated Innovation Strategy” (approved by the Cabinet on June 15, 2018). Cabinet Office, Government of Japan website (in Japanese) https://www8.cao.go.jp/cstp/tougosenryaku/tougo_honbun.pdf (accessed March 17, 2022).

4 “Bio Strategy 2019: Building a bio-community that resonates with Japanese and international communities” (approved by the Integrated Innovation Strategy Promotion Council on June 11, 2019). Cabinet Office, Government of Japan website (in Japanese) https://www8.cao.go.jp/cstp/bio/bio2019_honbun.pdf (accessed March 17, 2022).

5 “Handling of organisms obtained using genome editing technologies and not regarded as ‘living modified organism’ specified in the Cartagena Act” (Notification No. 1902081, issued by Nature Conservation Bureau, Ministry of the Environment, dated February 8, 2019). Ministry of the Environment website (in Japanese) https://www.env.go.jp/ press/20190208_shiryou1.pdf (accessed March 17, 2022).

6 “Food Hygiene Handling Procedures for Food and Additives Derived from Genome Editing Technology” (Decision by the Councillor for Environmental Health and Food Safety, Minister’s Secretariat dated September 19, 2019; last revised on December 23, 2020). Ministry of Health, Labour, and Welfare website (in Japanese) https://www.mhlw.go.jp/content/000709708.pdf (accessed March 17, 2022).

7 “Act on the Conservation and Sustainable Use of Biological Diversity through Regulations on the Use of Living Modified Organisms (Act No. 97 of 2003)”; enforced in 2004.

8 Japan Biosafety Clearing House (J-BCH) website (in Japanese) http://www.biodic.go.jp/bch/bch_8.html (accessed March 17, 2022).

9 “Concrete procedures for providing information, etc. on effects of organisms obtained using genome editing technologies on biodiversity in the field of agriculture, forestry, and fisheries,” The products information is also provided on the website. MAFF website (in Japanese) https://www.maff.go.jp/j/syouan/nouan/carta/tetuduki/nbt_tetuzuki.html (accessed March 17, 2022).

10 Animal feeds are subject to the Act on Safety Assurance and Quality Improvement of Feeds (Act No. 35 of 1953). MAFF is responsible for the feed and feed additives.

11 Although the notification procedure is not a legal requirement, it is also important to understand that in Japan what is asked by the government is usually something that must be followed, even if it is not required by law. Government and society thus expect that these procedures would be followed. MHLW states in their handling procedure, if there are cases found not following the notification process, there might be a case where the information of such developers are disclosed to public.

12 “A list of foods and food additives for which a notification has been completed according to the Food Hygiene Handling Procedures for Food and Additives Derived from Genome Editing Technology,” Ministry of Health, Labour, and Welfare website (in Japanese) https://www.mhlw.go.jp/stf/seisakunitsuite/bunya/kenkou_iryou/shokuhin/bio/genomed/newpage_00010.html (accessed March 17, 2022).

13 MHLW also decided in 2020 that the crossbred progeny of genome edited products will not be subject to regulation and no prior consultation/notification is needed. Ministry of Health, Labour, and Welfare website (in Japanese) https://www.mhlw.go.jp/content/000709708.pdf (accessed March 17, 2022).

14 Certain food items, such as oil and soy sauce, and animal feeds in which genetically modified DNA or resulting proteins are not identified are exempt from labeling.

15 Food Labeling Division, Consumer Affairs Agency, “Labeling of foods derived from genome editing technology,” September 2019. Consumer Affairs Agency website (in Japanese) https://www.caa.go.jp/policies/policy/food_labeling/quality/genome/pdf/genome_190919_0001.pdf (accessed March 17, 2022). “Labeling information for foods derived from genome editing technology”, Consumer Affairs Agency website (in Japanese) https://www.caa.go.jp/policies/policy/food_labeling/quality/genome/ (accessed March 17, 2022).

16 “Issues concerning foods derived from genome editing technology (Appendix to “Frequently asked questions about the Food Labeling Standards” (Notification No. 140, issued by Food Labeling Division, Consumer Affairs Agency, dated March 30, 2015)”. Consumer Affairs Agency website (in Japanese) https://www.caa.go.jp/policies/policy/food_labeling/food_labeling_act/pdf/food_labeling_act_190919_0011.pdf (accessed March 17, 2022).

17 Pioneer EcoScience Co., Ltd. website (in Japanese) https://p-e-s.co.jp/aozora (accessed March 17, 2022).

18 “Sales Launch of Genome-Edited Tomato Seedlings for Home Gardening”, Sanatech-seed. Sanatec-seed website (in Japanese) https://sanatech-seed.com/ja/211011/ (accessed March 17, 2022).

19 “We want to bring the world first genome edited “22nd Century Red Seabream,” to as many people as possible!,” Campfire website (in Japanese), https://camp-fire.jp/projects/view/400934?list=search_result_projects_popular (accessed March 17, 2022).

20 Biostation website (in Japanese), https://bio-sta.jp/news/administration/2750/ (accessed March 17, 2022).

21 “We want to bring the environment friendly genome edited “22nd Century tiger puffer,” to as many people as possible!,” Campfire website (in Japanese), https://camp-fire.jp/projects/view/512578?list=popular (accessed March 17, 2022).

22 Campfire website (in Japanese), https://camp-fire.jp/projects/view/512578?list=popular (accessed March 17, 2022).

23 “List of products”, Regional Fish website (in Japanese) https://regionalfish.online/collections/frontpage (accessed on 2nd April, 2022).

24 There were four items in the questionnaire; “Strict regulations should be put in place to minimize risk to as close to zero as possible;” “Regulations should be based on scientifically and technologically sound standards;” “Emphasis should be placed on economic efficiency, and regulations should be based on the minimum necessary standards;” and “There is no need to regulate”.

25 Such as the “No GMO Campaign” to gather petition signatures. There were also other movements to label products as “OK Seed” that did not use genome editing.

26 One factor may be that opposition groups that used to be influential are seeing their members become increasingly older with fewer younger generations joining.

27 “Home gardeners can expect to pick the world's first genome-edited tomato from Sanatech Seed in their garden in June.” Nikkei Biotechnology & Business website (in Japanese) https://bio.nikkeibp.co.jp/atcl/news/p1/21/04/28/08105/ (accessed March 17, 2022).

28 Bio-Station website (in Japanese) https://bio-sta.jp/ (accessed March 17, 2022).

29 “[Part 2] License to use patented inventions” Bio-Station website (in Japanese) https://bio-sta.jp/development/2310/ (accessed March 17, 2022).

30 For the detailed discussions on the process of clarifying the regulations in Japan and what characterizes the process, see Matsuo (2021).

31 In this paper we will not go into detail, but there are differences in the scope of the regulated products for the environment and for food use; for the environment (i.e., impact on biodiversity) aspects, decisions were made in accordance with the Cartagena Act and using the regulatory triggers strictly as defined in the existing laws and regulations, whereas the food safety aspects were dealt with on a risk-based manner, albeit based on the Food Sanitation Act. Since laws applied for environmental use and food use in Japan are different and regulators of MOE and MHLW differed in their approaches in the interpretation, theoretically, the exemption scope can be different depending on the product [i.e. SDN2 products – MOE treat it as LMOs (not exempted) but MHLW will decide after prior consultation, GM food or food for notification (exempted)].

32 In Europe, the European Court of Justice has ruled that organisms obtained by mutagenesis are GMOs within the meaning of the Directive on the release of GMOs into the environment, in so far as the techniques and methods used alter the genetic material of the organisms in a way that does not occur naturally and that those organisms are, in principle, subject to the obligations laid down by the Directive. However, revision of this approach is currently being considered. “Legislation for plants produced by certain new genomic techniques” EU website https://ec.europa.eu/info/law/better-regulation/have-your-say/initiatives/13119-Legislation-for-plants-produced-by-certain-new-genomic-techniques_en (accessed April 3, 2022).

33 Those are made available on the MOE's Japan Biosafety Clearing-House (J-BCH) website and the MHLW website.

34 Pioneer EcoScience Co., ltd website (in Japanese) https://p-e-s.co.jp/store/products/list?category_id=2 (accessed March 17, 2022).

35 Initiatives aimed at promoting consumer understanding include the Network for Breeding by Genome Editing, which was established by the University of Tsukuba as a leading member and with a cooperation of those concerned in genome editing breeding with backgrounds in industry, academia, and media. Network for Breeding by Genome Editing website (in Japanese) https://genome.t-pirc.tsukuba.ac.jp/network (accessed March 17, 2022).

36 “Aozora Tomato Gakuen” Pioneer EcoScience Co., Ltd. website (in Japanese) https://p-e-s.co.jp/aozora (accessed March 17, 2022).

37 “Informing the company policy on labeling and traceability in order to provide genome edited “22^nd^ Century red seabream,” Regional Fish website (in Japanese) https://regional.fish/projects/post-4751/ (accessed March 17, 2022). The company has built a system that allows actual users to trace the production and processing history of its products using a QR code.

38 “Regional Fish, a start-up known for ultra-fast breeding and smart aquaculture, raises 400 million yen in capital by third-party allotment.” PR TIMES website (in Japanese) https://prtimes.jp/main/html/rd/p/000000003.000060432.html (accessed March 17, 2022). “Providing a “rental transfer” to the MAFF to develop our country by human resource development in food tech and regional development ventures.” PR TIMES website (in Japanese) https://prtimes.jp/main/html/rd/p/000000045.000015387.html?fbclid=IwAR1np7xrqdDlqKrdu9vIPLLnJ2y-xfGXDom8n3ER5dpqLstpf86o1z4LXz4 (accessed April 17, 2022).

39 “Initiating the “rental transfer” from the MAFF” Regional Fish website (in Japanese) https://regional.fish/projects/post-4718/ (accessed March 17, 2022).

40 Regional Fish website (in Japanese) https://regional.fish/ (accessed March 17, 2022).

41 “Transform SDG 2030, SDGs awareness survey” The Asahi Shimbun website (in Japanese), https://miraimedia.asahi.com/sdgs_survey08/ (accessed April 17, 2022).

42 “Develop sustainable industry by food tech” MAFF website (in Japanese) https://www.maff.go.jp/j/kanbo/foodtech/kenkyukai.html (accessed March 17, 2022).

43 Examples of university start-ups include PtBio, Inc. (Hiroshima University); EdiGene Inc. (the University of Tokyo); Bio Palette Co., Ltd. (Kobe University) and; EditForce, Inc. (Kyushu University).
