# Supplementary material for: Implications and Lessons From the Introduction of Genome-Edited Food Products in Japan
Source: Front Genome Ed. 2022 Jun 21;4:899154. doi: 10.3389/fgeed.2022.899154 (PMC9258185; doi:10.3389/fgeed.2022.899154)
Supplement: Supplementary file 1 [file DataSheet1.pdf]

Annex

List of information to be provided and disclosed for environmental release and food derived from genome-editing. Below are tentative translations by the authors of the information. Please refer to original source for reference.

Annex Tables

Annex table 1. List of information to be provided to the MOE

(a) The organism does not possess remnants of extracellularly processed nucleic acids or their replicated products, as stipulated in the Cartagena Act (including bases of decision)	
(b) Taxonomical species of the modified organism	Summary posted on J-BCH
(c) Method of genome editing used for modification	
(d) Modified gene and its functions	
(e) Trait change caused by the modification	Summary posted on J-BCH
(f) Other trait changes than those described in (e) Description of the changes, if any.	
(g) Usage of the organism	Summary posted on J-BCH
(h) Discussion of the organisms possible influence on biological diversity.	Summary posted on J-BCH

Source: Notification by the MOE concerning the handling of organisms not subject to the Cartagena Act[1]

Annex table 2. List of information to be provided to FSCAB/MAFF concerning agricultural products for environmental release

1 Name and description of the organism obtained using genome editing technologies

[1] "Handling of organisms obtained using genome editing technologies and not regarded as "living modified organism" specified in the Cartagena Act," MOE website (in Japanese) https://www.env.go.jp/press/20190208_shiryou1.pdf (accessed April 17, 2022)

2 Usage of the organism	
3 Description of the facility used	
4 The organism does not possess nucleic acids or their replicated products as a result of using technologies for extracellularly processing nucleic acids, as stipulated in Article 2, paragraph 2 (2), item (1) of the Cartagena Act	(1) Were extracellularly processed nucleic acids inserted (including information on the nucleic acids inserted)
	(2) Were there remnants of the inserted nucleic acids (including information on the selection/growth process and the method by which this was confirmed.)
5 Taxonomical species of the modified organism	(1) Name of the taxonomical species and variety or line of the host
	(2) Distribution status, history, and current use situation, as well as the physiological and ecological properties in the natural environment
6 Method of genome editing used for modification	(1) Information on artificial nuclease used
	(2) Method of introducing the artificial nuclease
7 Modified gene and its functions	(1) Genomic region of the host that was cleaved, for example, as a target, and changes in the region
	(2) Information regarding the gene targeted and changes in traits that could theoretically be caused by the modification
8 Changes in the traits introduced by the modification	
9 Other changes in the traits than those described in item 8. Please describe the changes, if any.	(1) Information regarding the possibility of modification of the non-target region
	(2) Other changes in traits than those described in item 8 in the organism created when compared to the host
10 Discussion of the organisms possible influence on biological diversity	(1) Competitive advantage<br>(2) Predatory or parasitic properties<br>(3) Production of harmful substances

	(4) Crossing properties<br>(5) Other characteristics<br>(6) Comprehensive discussion

Source: Procedures for providing information, etc. on biodiversity effects in the field of agriculture, forestry, and fisheries[2]

Annex table 3. Information to be noted and published on the website regarding food/additives derived from genome editing technologies

Food derived using genome editing technology	Information to be notified to the MHLW	(i) Name of the item, breed, and summary (usage and intended use) of the developed food<br>(ii) Genome editing technology used and modification details<br>(iii) Confirmation that foreign genes or their parts do not persist<br>(iv) Confirmation that the confirmed changes in the DNA do not cause the production of new allergens with adverse effects on human health or increase the levels of known toxic substances<br>(v) Changes in the major components (nutrient components only) related to the target metabolic system and modifications which were performed to affect the metabolic system and increase or decrease specific components<br>(vi) Year and month of marketing (*Notify the MHLW of it after marketing)
	Information published by the MHLW	(i) Names of the notifier and developer, and date (year/month/day) of notification<br>(ii) Name of item, breed, and summary (usage and intended use)<br>(iii) Summary of the genome editing technology used and gene modification<br>(iv) Confirmation that the confirmed changes in DNA do not cause the production of new allergens that adversely affect human health or increase levels of known toxic substances<br>(v) Summary of changes in major components (nutrient components only) related to the target metabolic system<br>(vi) Year and month of marketing (*Publish it after receipt of notification mentioned in (vi) above)
Additive derived from genome	Information to be notified to the MHLW	(i) Name of the item and summary (usage and intended use) of the developed additive<br>(ii) Method of genome editing technology used and modification details<br>(iii) Confirmation that foreign genes or their parts do not persist in the product<br>(iv) The additive complies with the compositional standards specified

[2] "Concrete procedures for providing information, etc. on effects of organisms obtained using genome editing technologies on biodiversity in the field of agriculture, forestry, and fisheries (Notification of Director-General of Food Safety and Consumer Affairs Bureau, MAFF, FSCAB/MAFF Notification No. 2743 dated October 9, 2019), " MAFF website (in Japanese) https://www.maff.go.jp/j/syouan/nouan/carta/tetuduki/attach/pdf/nbt_tetuzuki-8.pdf (accessed April 17, 2022)

editing technology		in the Notification on the Specifications and Standards<br>(v) Year and month of marketing (*Notify the MHLW of it after marketing)
	Information published by the MHLW	(i) Names of the notifier and developer, and date (year/month/day) of notification<br>(ii) Name of the item<br>(iii) Summary of the genome editing technology and gene modification<br>(iv) The additive complies with the compositional standards specified in the Notification on the specifications and standards<br>(v) Year and month of marketing (*Publish it after receipt of notification mentioned in (v) above)

Source: Table created using the Food Hygiene Handling Procedures for Food and Additives Derived from Genome Editing Technology[3]

[3] "Food Hygiene Handling Procedures for Food and Additives Derived from Genome Editing Technology (Decision by the Councilor for Environmental Health and Food Safety, Minister's Secretariat dated September 19, 2019)," MHLW website (in Japanese) https://www.mhlw.go.jp/content/000709708.pdf (accessed April 17, 2022)
